# Does subnetting and port hardening influence human adversarial decisions? An investigation via a HackIT tool

**DOI:** 10.3389/fdata.2023.988007

**Published:** 2023-06-15

**Authors:** Shashank Uttrani, Palvi Aggarwal, Varun Dutt

**Affiliations:** ^1^Applied Cognitive Science Lab, Indian Institute of Technology, Mandi, India; ^2^Department of Computer Science, University of Texas El Paso, El Paso, TX, United States

**Keywords:** cybersecurity, deception, hacking, HackIT tool, honeypot, port hardening, subnetting

## Abstract

Prior research in cyber deception has investigated the effectiveness of the timing of deception on human decisions using simulation tools. However, there exists a gap in the literature on how the availability of subnets and port-hardening influence human decisions to attack a system. We tested the influence of subnets and port-hardening on human attack decisions in a simulated environment using the HackIT tool. Availability of subnets (present/absent) within a network and port-hardening (easy-to-attack/hard-to-attack) were varied across four between-subject conditions (*N* = 30 in each condition): with-subnet with easy-to-attack, with-subnet with hard-to-attack, without-subnet with easy-to-attack, and without-subnet with hard-to-attack. In with-subnet conditions, 40 systems were connected in a hybrid topology network with ten subnets connected linearly, and each subnet contained four connected systems. In without-subnet conditions, all 40 systems were connected in a bus topology. In hard-to-attack (easy-to-attack) conditions, the probabilities of successfully attacking real systems and honeypots were kept low (high) and high (low), respectively. In an experiment, human participants were randomly assigned to one of the four conditions to attack as many real systems as possible and steal credit card information. Results revealed a significant decrease in the proportion of real system attacks in the availability of subnetting and port hardening within the network. Also, more honeypots were attacked in with-subnet conditions than without-subnet conditions. Moreover, a significantly lower proportion of real systems were attacked in the port-hardened condition. This research highlights the implications of subnetting and port-hardening with honeypots to reduce real system attacks. These findings are relevant in developing advanced intrusion detection systems trained on hackers' behavior.

## Introduction

There has been an ever-increasing concern related to information security in this age of digital connectivity (Jackson, 2015). Cyber-attackers have surreptitiously been trying to gain access to networks containing relevant information not just by conventional means of hacking but also by malicious activities such as phishing (Silic and Back, 2016). Instead of traditional methods of prevention against such attacks, more aggressive forms of defense options are now being put into place, such as using deception via honeypots (Araujo et al., 2018; Sutton et al., 2019; Aggarwal et al., 2020; Katakwar et al., 2020).

Honeypots are fake systems introduced within the network to lure hackers (Spitzner, 2003; Mohammed and Pathan, 2013). These systems may contain information that seems real and valuable to attackers (Spitzner, 2003; Mohammed and Pathan, 2013; Kambow and Passi, 2014). Research in the area of the use of honeypots has been successful in identifying how honeypots can be created and deployed (Kambow and Passi, 2014). Also, behavioral cybersecurity researchers have extensively investigated honeypots' use in predicting adversary's decisions during a cyber-attack (Furman et al., 2012; Addae et al., 2016; Caulkins et al., 2016). Researchers have documented the effect of early and late deception on an attacker's decision using abstract games and simulated networks (Singal et al., 2017; Aggarwal et al., 2020). Additionally, the effect of network sizes (Katakwar et al., 2020), rewards (Chatfield and Reddick, 2017), and punishments (Maqbool et al., 2020) associated with attacks have been elucidated in the recent past. Research has also investigated the effects of the network's different topologies on the adversary's attacking decisions (La, 2014; Achleitner et al., 2017; Kelly et al., 2019).

Different network topologies have been investigated to capture their effects on adversary behavior during a cyber-attack (Chadha et al., 2016; Veksler et al., 2018). For example, Achleitner et al. (2017) investigated the influence of the presence and absence of subnetting in networks using Software-Defined Networking (SDN) based virtual topologies. These researchers observed a decrease in the proportion of systems attacked in networks when subnets were present compared to networks where subnets were absent (Achleitner et al., 2017). Similar results were reported by Kelly et al. (2019) in their investigation of the influence of subnets' availability during cyber-attacks. These researchers modeled the adversarial network environment again using SDN-based virtual topologies (Kelly et al., 2019).

Furthermore, various system hardening tools and techniques have been employed to reduce the risk of exploiting system vulnerabilities (Nguyen-Tuong et al., 2005; Turnbull, 2005). The objective of these tools is to condense the attacking surface on a system. The reduction of attacking surface can be made by introducing access limitations at various levels such as application level, network level, or firmware level. One such technique is port hardening (Gunes et al., 2021) which refers to the process of securing network ports to prevent unauthorized access, protect the system's integrity, and reduce the risk of cyber-attacks. It involves configuring network ports to limit access to the resources and services on a network. Prior research has investigated the influence of port hardening on adversary behavior and reported a decrease in the proportion of systems attacked when port hardening is present (Albanese et al., 2012; Dietz and Wallach, 2014). However, little is known about the influence of port hardening in networks on adversarial decision-making during cyber-attacks.

Aggarwal et al. (2016) and Katakwar et al. (2020) investigated adversary's behavior against different network sizes and found that medium and large-sized networks are attacked more than small-sized networks in the presence of honeypots. Similarly, Achleitner et al. (2017) and Kelly et al. (2019) showed improved resilience and reduced the success rate of cyber-attacks in the presence of subnetting, which required dividing the network computers into smaller network clusters. However, little is known about how the availability of subnets and port hardening influence adversarial decisions in networks containing honeypots. This research's primary objective is to address these literature gaps and investigate the influence of the availability of subnets within a network and the port hardening of computer systems on adversarial decisions. Specifically, we plan to evaluate the proportion of real and honeypot systems attacked when adversaries are presented with networks configured with different network topologies (presence and absence of subnets) and different levels of port hardening (easy-to-attack and hard-to-attack). In the with-subnet configurations, all the systems were connected in a hybrid topology (a combination of star and linear topology). In the without-subnet configurations, all systems are connected in a bus topology. In port hardening, the probability of attacking a system is kept very high in an easy-to-attack configuration compared to the hard-to-attack configuration.

Some of the key contributions of this research can be encapsulated in the following points.

- First, the suggested investigations in this paper would help provide theoretical and practical advancements in understanding adversarial behavior during a cyber-attack under the influence of subnets' availability within a network and port hardening of computer systems.
- Second, such an investigation may also help the adversarial cybersecurity and cognitive science communities to develop models that could help network architects and network designers test network security and reduce the damage occurring due to cyber-attacks.
- Third, classification between a genuine user and a hacker may also be made possible by utilizing the data collected on behavioral patterns of accessing networked systems and the services running on the ports of these systems.

Overall, this research's main novelty is the investigation of combined effect of availability of subnets and port hardening on adversary's decision during a cyber-attack.

In what follows, first, we recap the literature on the attacking behavior of adversaries under the influence of the availability of subnets within a network and the port hardening of computer systems. Next, we present a laboratory experiment involving human participants tasked to attack a network of web servers configured using a real-time simulation tool called HackIT (Aggarwal et al., 2020). Finally, we close the paper by discussing the implications of our results.

## Related work

Prior research in network security has documented adversaries' attacking behavior in different network topologies presence and absence of subnets within a network (Achleitner et al., 2017; Kelly et al., 2019). In the absence of subnets, the network contains all the systems connected in a bus topology. In contrast, in the presence of subnets, the network contains various subnets within the network, which are connected. There is complete visibility of the network structure and its nodes (or systems) in without-subnet conditions. In the with-subnet conditions, only the nodes connected within a subnet are visible. The main findings are that a higher proportion of systems are attacked in without-subnet conditions (Webster et al., 2006; Pu and Faltings, 2011). However, in with-subnet conditions, a smaller proportion of systems are attacked (Webster et al., 2006; Pu and Faltings, 2011). The decrease in the proportion of attacks may be due to the limited and hindered visibility of the complete network structure. Such limited and hindered visibility may be due to the adversary's bounded rationality, which is governed by cognitive theories like Instance-based Learning (Gonzalez et al., 2003; Dutt and Gonzalez, 2012). Overall, there is likely an influence of the availability of subnets within a network on the proportion of systems attacked, and we hypothesize that:

*H1:* The proportion of systems attacked will be smaller when subnets are present within a network compared to when subnets are absent.

Literature in adversarial cybersecurity research has documented the influence of system hardening on adversaries' decisions during cyber-attacks, i.e., when the ports of systems are hard-to-attack and easy-to-attack (Albanese et al., 2012). In the hard-to-attack condition, the ports of systems in a network are difficult to access. In an easy-to-attack condition, the ports of systems in a network are easily accessible. Results show that when a hard-to-attack condition is implemented in a network, the proportion of system attacks is smaller than when an easy-to-attack condition is implemented in a network (Nguyen-Tuong et al., 2005; Turnbull, 2005). The decrease in the proportion of attacks may be due to the limited accessibility of systems to adversaries and the limited sample size when trying to probe and attack different systems. Overall, there is an effect of port hardening of systems within a network on the proportion of systems attacked, and we hypothesize that:

*H2:* The proportion of systems attacked will be smaller in hard-to-attack (port-hardened) conditions than easy-to-attack (non-port-hardened) conditions.

Research in adversarial cybersecurity has also documented the influence of time on adversaries' attacking behavior during a cyber-attack (Achleitner et al., 2017; Kelly et al., 2019). Results show a significant increase in the proportion of systems attacked over time (Achleitner et al., 2017; Kelly et al., 2019). This increase in system attacks has been attributed to the fact that adversaries gain familiarity with the network structure over some time and tend to probe and attack systems in a meditated method (Webster et al., 2006; Pu and Faltings, 2011). Overall, there is an effect of time on the proportion of systems attacked within a network, and we hypothesize that:

*H3:* The proportion of systems attacked will increase over time.

Although the main effects of the availability of subnets within a network and port hardening of systems in a network have been investigated, however little is known about the combined effect of the availability of subnets within a network and port hardening of systems in a network on adversaries' attacking behavior during a cyber-attack (Albanese et al., 2012; La, 2014; Achleitner et al., 2017; Kelly et al., 2019). According to the literature on adversarial cybersecurity, a lower proportion of systems are attacked in a network when there is a presence of subnets within the network than when there is an absence of subnets within a network. Similarly, according to the literature, a lower proportion of systems are attacked when some kind of system hardening (such as port hardening) is implemented on systems in a network compared to when no system hardening is implemented on systems in the network (Nguyen-Tuong et al., 2005; Turnbull, 2005; Dietz and Wallach, 2014). Therefore, we expect a higher proportion of attacks on systems in conditions where the subnet is absent and real systems are easy-to-attack. Thus, we hypothesize that:

*H4:* The disparity in the number of systems targeted during attacks will be more pronounced when real systems are easily attacked compared to when they are difficult to attack, particularly in scenarios where subnets are not present compared to scenarios where subnets are present.

Furthermore, previous research in adversarial cybersecurity has documented the main effects of port hardening on adversaries' attacking decisions during a cyber-attack (Dietz and Wallach, 2014). However, little is known about the effects of port hardening of systems in a network over time on adversaries' attack behavior. The result of port hardening's main effect shows that there is an increase in the proportion of systems attacked when an easy-to-attack configuration is adopted compared to a hard-to-attack configuration (Nguyen-Tuong et al., 2005; Turnbull, 2005; Dietz and Wallach, 2014). Also, there is an increase in the proportion of systems attacked over time, as per the literature (Kelly et al., 2019). Thus, we hypothesize that:

*H5:* There will be a significant increase in the difference over time between proportion of real and honeypot systems attack when real systems are easy to attack and when real systems are hard to attack.

Moreover, the combined effect of subnets' availability over time within a network and port hardening of systems over time on adversaries' attacking behavior has not been investigated yet. As per the literature on the main effects of subnet availability (Albanese et al., 2012; La, 2014; Achleitner et al., 2017; Kelly et al., 2019) and port hardening (Nguyen-Tuong et al., 2005; Turnbull, 2005; Dietz and Wallach, 2014) on adversaries' attacking behavior over time, we hypothesize that:

*H6:* The proportion of systems attacked over time will be more significant in the without-subnet condition than the with-subnet condition in the easy-to-attack configuration. However, in hard-to-attack conditions, the proportion of systems attacked over time will be more significant in the with-subnet condition than the without-subnet condition.

## Experiment

We performed a laboratory experiment involving human participants, performing as hackers, to investigate the influence of subnets and port hardening during cyber-attacks using the HackIT tool. In the experiment, we compared the proportion of real and honeypot systems attacked in the presence and absence of subnets within a network with the varying hardness of ports of systems present. Based on the prior literature (Achleitner et al., 2017; Aggarwal et al., 2020), we expected a higher proportion of real system attacks when no subnets were present within the network compared to when subnets were present within the network. Furthermore, we expected a lower proportion of system (real and honeypot) attacks in conditions where ports were hard to attack than in conditions where ports were easy to attack.

## Methods

### Participants

A total of 300 participants were anonymously recruited through Amazon Mechanical Turk, a crowdsourcing website (Mason and Suri, 2012), to participate in an online cybersecurity study using the HackIT tool. The Ethics Committee approved the research at the Indian Institute of Technology Mandi. Participation was voluntary, and all participants gave written consent before starting their study. All participants were screened through a series of questions from networking and cybersecurity areas before starting the study. One hundred twenty participants cleared the screening test with 70% or more correct choices. A total of 10 questions of approximately similar difficulty, covering theoretical and practical topics of computer networks, data transmission, and network security, were used in the screening test to test participants' domain knowledge. The maximum time duration allotted for screening test was 15 min. Successful recruits were allowed to continue with the study, and the rest were thanked for their participation. Seventy-eight percent of participants who cleared the screening test were male, and the rest were females. The participants ranged between 19 years and 58 years of age (median = 31 years, mean = 32, and standard deviation = 6.7 years). Around 72.5% of participants possessed a bachelor's degree, while 27.5% possessed a master's or a doctoral degree. Also, 65% of the participants had a degree with computer science as a major, 6% had electrical engineering as a major, and the remaining participant had a degree with basic sciences and management subjects as a major. All the participants had taken a course in computer networks or network security in the past. Participants were reimbursed INR 50 (USD 0.67) after they completed their study successfully. Also, there was a performance incentive based upon a lucky draw. The top-3 scorers of the study were put in a lucky draw, and one of the participants was randomly selected and awarded a gift voucher of INR 500 (USD 6.67).

### Experimental design

**Figure 1:**
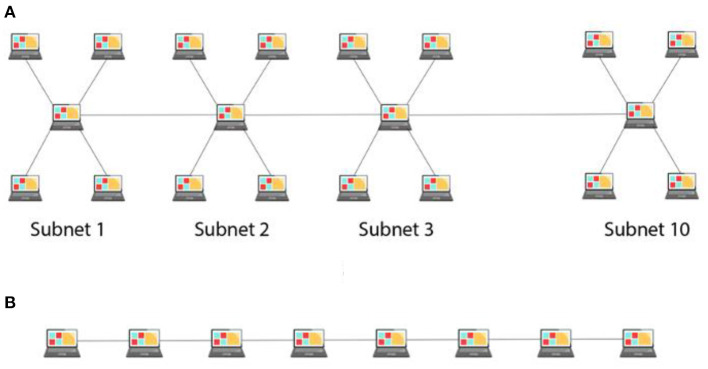
**(A)** An example of hybrid topology in a network (with-subnet condition) and **(B)** an example of linear or bus topology in a network (without-subnet condition).

Participants were randomly assigned to one of the four between-subject conditions (N = 30 in each condition): with-subnet and easy-to-attack (WSE), with-subnet and hard-to-attack (WSH), without-subnet and easy-to-attack (WoSE), and without-subnet and hard-to-attack (WoSH). The HackIT tool was configured according to the condition assigned to the participant. In with-subnet conditions (WSE and WSH), 40 systems (consisting of 30 honeypot systems and 10 real systems) were connected in a hybrid integration of star and bus topology. Figure 1A illustrates the arrangement of nodes (or systems) in with-subnet conditions. There were 10 subnets within the network, and each subnet consisted of 4 systems connected in a star topology. The 10 subnets were then connected in a bus topology via the hub systems of each subnet. In without-subnet conditions (WoSE and WoSH), however, all 40 systems were connected linearly in a bus topology, as shown in Figure 1B.

Since port hardening can be achieved by closing unnecessary ports, configuring firewalls, and using access control lists (ACLs), two conditions were designed with different access probabilities of ports on each system. In hard-to-attack conditions (WSH and WoSH), ports of systems were hardened by keeping the probability of attacking a system successfully at 0.1 in the HackIT tool. In contrast, the probability of attacking a system successfully was kept at 0.9 in the HackIT tool in easy-to-attack conditions (WSE and WoSE), thereby reducing ports' hardness. The participants' objective was to maximize their score by attacking as many real systems as possible in 10 min. Attacking a system meant exploiting a vulnerability in the system and stealing a credit card information file stored in all the systems, real as well as honeypots. The ratio of real systems to honeypot systems was kept the same across all four conditions; however, this information was not revealed to participants.

The simulated network environment (HackIT) consisted of 40 systems (10 real systems and 30 honeypot systems) having different operating systems and open ports on each system. Table 1 shows all the operating systems and available ports on the systems with services running on each port and their associated vulnerability to attack.

**Table 1:** List of different types of systems, operating systems, ports, services, and vulnerabilities defined within the simulated environment.

**System No**.	**Real or honeypot**	**OS**	**Ports**	**Services**	**Vulnerabilities**
1	Honeypot	Solaris	25/tcp, 21/tcp, 111/tcp, 135/tcp	smtp, ftp, rpcbind, msrpc	directory_harvest, brute_force, DDoS_attack, DoS_attack
2	Honeypot	HP-UX 11i	135/tcp, 111/tcp, 25/tcp, 80/tcp	msrpc, rpcbind, smtp, http	DoS_attack, DDoS_attack, directory_harvest, sql_injection
3	Honeypot	HP-UX 11i	80/tcp, 111/tcp, 21/tcp, 25/tcp	http, rpcbind, ftp, smtp	sql_injection, DDoS_attack, brute_force, directory_harvest
4	Honeypot	Windows Server 2003	111/tcp, 21/tcp, 135/tcp, 80/tcp	rpcbind, ftp, msrpc, http	DDoS_attack, brute_force, DoS_attack, sql_injection
5	Honeypot	HP-UX 11i	80/tcp, 21/tcp, 111/tcp, 25/tcp	http, ftp, rpcbind, smtp	sql_injection, brute_force, DDoS_attack, directory_harvest
6	Honeypot	Windows Server 2003	135/tcp, 80/tcp, 111/tcp, 21/tcp	msrpc, http, rpcbind, ftp	DoS_attack, sql_injection, DDoS_attack, brute_force
7	Honeypot	HP-UX 11i	80/tcp, 25/tcp, 21/tcp, 111/tcp	http, smtp, ftp, rpcbind	sql_injection, directory_harvest, brute_force, DDoS_attack
8	Honeypot	HP-UX 11i	135/tcp, 111/tcp, 25/tcp, 21/tcp	msrpc, rpcbind, smtp, ftp	DoS_attack, DDoS_attack, directory_harvest, brute_force
9	Honeypot	Solaris	21/tcp, 111/tcp, 25/tcp, 135/tcp	ftp, rpcbind, smtp, msrpc	brute_force, DDoS_attack, directory_harvest, DoS_attack
10	Honeypot	HP-UX 11i	135/tcp, 111/tcp, 21/tcp, 80/tcp	msrpc, rpcbind, ftp, http	DoS_attack, DDoS_attack, brute_force, sql_injection
11	Real	OpenBSD	443/tcp, 53/tcp, 3,306/tcp, 8,080/tcp	https, domain, MySql, apache	drown_attack, DNS_zone_transfer, remove_auth, url_decoder
12	Honeypot	HP-UX 11i	111/tcp, 135/tcp, 21/tcp, 80/tcp	rpcbind, msrpc, ftp, http	DDoS_attack, DoS_attack, brute_force, sql_injection
13	Honeypot	Windows XP	135/tcp, 21/tcp, 111/tcp, 80/tcp	msrpc, ftp, rpcbind, http	DoS_attack, brute_force, DDoS_attack, sql_injection
14	Honeypot	HP-UX 11i	135/tcp, 21/tcp, 111/tcp, 25/tcp	msrpc, ftp, rpcbind, smtp	DoS_attack, brute_force, DDoS_attack, directory_harvest
15	Honeypot	Windows XP	25/tcp, 80/tcp, 111/tcp, 21/tcp	smtp, http, rpcbind, ftp	directory_harvest, sql_injection, DDoS_attack, brute_force
16	Real	Linux	443/tcp, 110/tcp, 53/tcp, 5,800/tcp	https, pop3, domain, vncc http	drown_attack, pop3_version, DNS_zone_transfer, remote_auth
17	Honeypot	Windows Server 2003	111/tcp, 21/tcp, 25/tcp, 135/tcp	rpcbind, ftp, smtp, msrpc	DDoS_attack, brute_force, directory_harvest, DoS_attack
18	Real	Windows 8	110/tcp, 139/tcp, 5,800/tcp, 3,306/tcp	pop3, netbios-ssn, vncc http, MySql	pop3_version, DCOM_buffer_overrun, remote_auth, remove_auth
19	Honeypot	Solaris	80/tcp, 111/tcp, 135/tcp, 21/tcp	http, rpcbind, msrpc, ftp	sql_injection, DDoS_attack, DoS_attack, brute_force
20	Real	Linux	445/tcp, 3,306/tcp, 139/tcp, 53/tcp	microsoft-ds, MySql, netbios-ssn, domain	windows_null_session, remove_auth, DCOM_buffer_overrun, DNS_zone_transfer
21	Honeypot	Solaris	25/tcp, 80/tcp, 135/tcp, 21/tcp	smtp, http, msrpc, ftp	directory_harvest, sql_injection, DoS_attack, brute_force
22	Honeypot	HP-UX 11i	111/tcp, 80/tcp, 25/tcp, 21/tcp	rpcbind, http, smtp, ftp	DDoS_attack, sql_injection, directory_harvest, brute_force
23	Honeypot	Solaris	21/tcp, 25/tcp, 80/tcp, 135/tcp	ftp, smtp, http, msrpc	brute_force, directory_harvest, sql_injection, DoS_attack
24	Real	Mac OS X	443/tcp, 445/tcp, 6,112/tcp, 139/tcp	https, microsoft-ds, dtspc, netbios-ssn	drown_attack, windows_null_session, remote_exploit_buffer_overflow, DCOM_buffer_overrun
25	Honeypot	Solaris	111/tcp, 21/tcp, 25/tcp, 80/tcp	rpcbind, ftp, smtp, http	DDoS_attack, brute_force, directory_harvest, sql_injection
26	Honeypot	Windows Server 2003	21/tcp, 135/tcp, 80/tcp, 111/tcp	ftp, msrpc, http, rpcbind	brute_force, DoS_attack, sql_injection, DDoS_attack
27	Honeypot	HP-UX 11i	135/tcp, 25/tcp, 111/tcp, 21/tcp	msrpc, smtp, rpcbind, ftp	DoS_attack, directory_harvest, DDoS_attack, brute_force
28	Real	Windows 8	53/tcp, 5,900/tcp, 22/tcp, 445/tcp	domain, vncc http, ssh, microsoft-ds	DNS_zone_transfer, remote_auth, user_auth, windows_null_session
29	Real	OpenBSD	6112/tcp, 53/tcp, 5,800/tcp, 22/tcp	dtspc, domain, vncc http, ssh	remote_exploit_buffer_overflow, DNS_zone_transfer, remote_auth, user_auth
30	Honeypot	HP-UX 11i	135/tcp, 25/tcp, 111/tcp, 21/tcp	msrpc, smtp, rpcbind, ftp	DoS_attack, directory_harvest, DDoS_attack, brute_force
31	Honeypot	HP-UX 11i	80/tcp, 111/tcp, 25/tcp, 21/tcp	http, rpcbind, smtp, ftp	sql_injection, DDoS_attack, directory_harvest, brute_force
32	Honeypot	Windows Server 2003	111/tcp, 135/tcp, 25/tcp, 80/tcp	rpcbind, msrpc, smtp, http	DDoS_attack, DoS_attack, directory_harvest, sql_injection
33	Real	Mac OS X	6,112/tcp, 445/tcp, 110/tcp, 139/tcp	dtspc, microsoft-ds, pop3, netbios-ssn	remote_exploit_buffer_overflow, windows_null_session, pop3_version, DCOM_buffer_overrun
34	Honeypot	Windows Server 2003	21/tcp, 135/tcp, 25/tcp, 111/tcp	ftp, msrpc, smtp, rpcbind	brute_force, DoS_attack, directory_harvest, DDoS_attack
35	Honeypot	HP-UX 11i	21/tcp, 135/tcp, 80/tcp, 111/tcp	ftp, msrpc, http, rpcbind	brute_force, DoS_attack, sql_injection, DDoS_attack
36	Real	Mac OS X	5,900/tcp, 53/tcp, 5,800/tcp, 6112/tcp	vncc http, domain, vncc http, dtspc	remote_auth, DNS_zone_transfer, remote_auth, remote_exploit_buffer_overflow
37	Honeypot	Windows Server 2003	135/tcp, 25/tcp, 21/tcp, 80/tcp	msrpc, smtp, ftp, http	DoS_attack, directory_harvest, brute_force, sql_injection
38	Real	OpenBSD	139/tcp, 443/tcp, 8,080/tcp, 5,900/tcp	netbios-ssn, https, apache, vncc http	DCOM_buffer_overrun, drown_attack, url_decoder, remote_auth
39	Honeypot	HP-UX 11i	135/tcp, 111/tcp, 21/tcp, 80/tcp	msrpc, rpcbind, ftp, http	DoS_attack, DDoS_attack, brute_force, sql_injection
40	Honeypot	Windows Server 2003	80/tcp, 135/tcp, 21/tcp, 111/tcp	http, msrpc, ftp, rpcbind	sql_injection, DoS_attack, brute_force, DDoS_attack

### Stimuli

**Figure 2:**
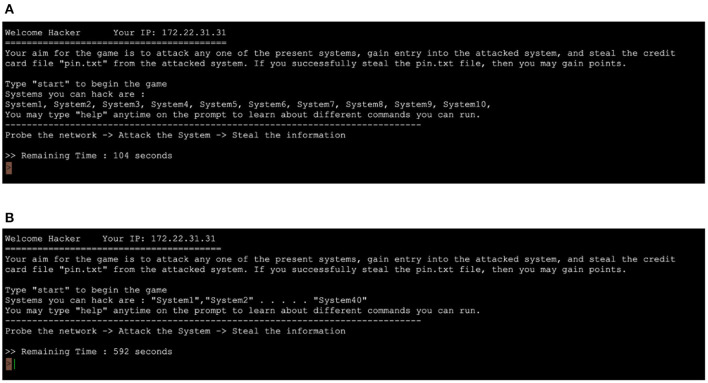
Initial instructions to the participants in **(A)** with-subnet condition and **(B)** without-subnet condition.

**Figure 3:**
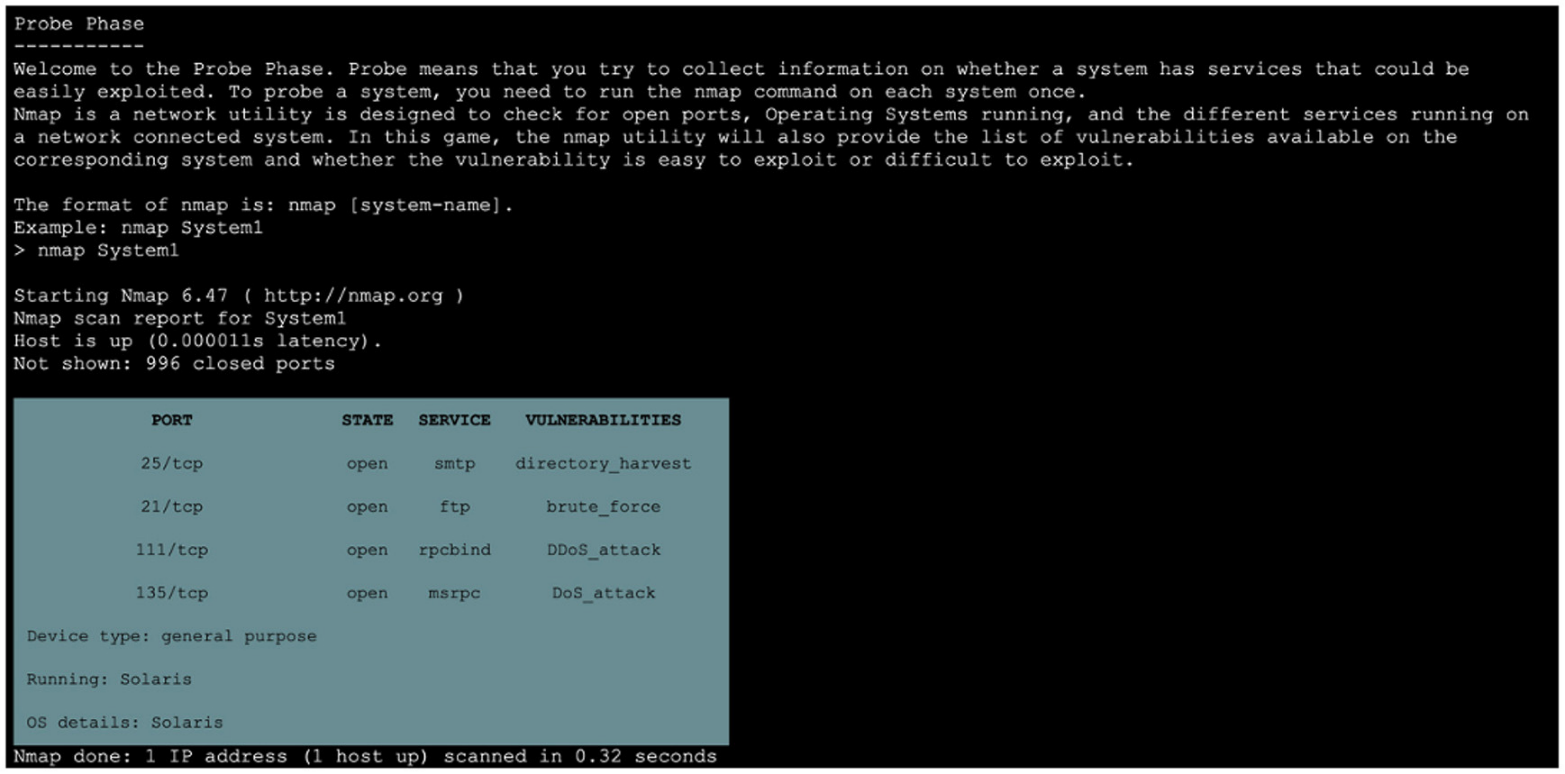
Demonstration of “nmap” command being used in probe phase.

For the experiment, we simulated a network of 40 systems using the HackIT tool for all four between-subject conditions. The recruited participants were tasked to hack the real systems by stealing a file containing credit card information located on those systems. The step-by-step HackIT task procedure is described as follows. Figures 2A, B show the objective and initial procedure presented to the participant once they signed into HackIT. The information on the available (or unhacked) systems was also presented to participants. The information related to each system, such as available ports, services, and vulnerability associated with those ports, were presented to the participant upon probing the system using the “nmap” command (see Figure 3). Participants used this information to decide whether to attack the probed system or not. To attack a system, the “use_exploit” command was used by participants after deciding the port and the vulnerability to attack in the system. The success of the attack depended upon the hardness associated with the system. If the attack was successful, participants could access the files present within the system using the “ls” command; otherwise, access was denied. Finally, the “scp” command was used by participants to remotely transfer the “pin.txt” file to their system. Once the transfer was complete, a feedback message was displayed whether the attacked system was a real system or a honeypot system. The reward for attacking the system was also displayed. Subsequently, a list of remaining systems available to probe and attack was displayed to participants. Participants' final score was displayed after the allotted time of 10 min had elapsed.

### Procedure

**Figure 4:**
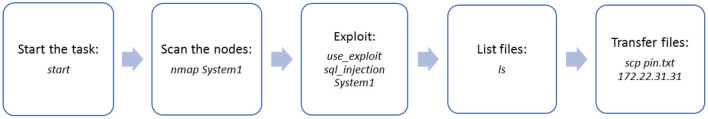
Flowchart of commands to be used during HackIT task.

The recruited participants were randomly assigned to one of four between-subject conditions via a weblink on the Amazon Mechanical Turk portal. Instructions related to the HackIT task's objective were given to participants before starting the task, along with a flowchart on how to proceed and use the available commands (see Figure 4). Specifically, the hackers' objective was to maximize their score by successfully hacking as many real systems present within the network and stealing the credit-card file (“pin.txt”) from those real systems within 10 min. Every attempt to hack a system involved two stages: The Probe stage and the Attack stage. In the Probe stage, participants performing as hackers could probe any number of systems using the “nmap” utility before attacking a system. After probing systems, participants performing as hackers received information about open ports, operating systems, services running, and vulnerabilities associated with those systems. Participants performing as hackers could use the presented information to decide whether a given system was real or not and whether to attack the corresponding system. Next, participants could attack the desired system using the “use_exploit” command. If a system got exploited, the hacker could search and transfer the “pin.txt” file to her remote system using the “scp” command to complete the hacking task. Otherwise, the hacker could exploit other available ports on that system or choose another system to probe or attack. Participants had 10 min to attack as many real systems as possible in HackIT. Once participants completed their study, they were thanked and paid for their participation.

### Data analysis

For analyzing data, a three-way mixed analysis of variance (ANOVA) was performed to evaluate our expectations. ANOVA is a statistical test to test the difference between two or more means across different conditions. The normality and homogeneity assumptions were tested using Q-Q plots and Levene's test (Field, 2013). The Q-Q plot confirmed the normal distribution of dependent variables (i.e., proportions of real systems attacked and honeypot systems attacked) in different conditions. Also, Levene's test revealed the variances to be homogenous for the proportion of real and honeypot systems across different conditions. It was advantageous to use ANOVA over other methods, such as non-parametric tests, because ANOVA provides the overall test of the quality of group means and controls the Type 1 error rates (i.e., false positive choices) (Field, 2013). ANOVA is also more powerful than non-parametric tests with the normal distribution of data (Field, 2013).

The F-statistic in ANOVAs represents the ratio of between-group variance and within-group variance (Weir and Hill, 2002; Field, 2013). F-statistics variables are the degrees of freedom (K-1, N-K), where K is the total number of groups compared, and N is the overall sample size. The *p*-value indicates the evidence in favor of the null hypothesis when it is true. We rejected the null hypothesis when the *p*-value was less than the alpha level (=0.05). The eta-square is the proportion of variance associated with one or more main effects. It was a number between 0 and 1. A value of 0.02, 0.13, and 0.26 measured small, medium, or large effect sizes (or correlations), respectively, between the dependent and independent variables given population size.

## Results

The mixed factorial ANOVA investigated the influence of availability of subnets within a network (between-subject factor), hardening of ports of systems (between-subject factor), and proportion of real and honeypot systems attacker over time (within-subject factor). The dependent variables used were the proportion of real system attacked and the proportion of honeypot systems attacked in the HackIT tool. The proportion of real system attacked, and the proportion of honeypot system attacked was computed at the following times during the experiment: 2 min, 4 min, 6 min, 8 min, and 10 min.

Furthermore, we analyzed the proportion of honeypots and real systems attacked using different ports available across different systems in the HackIT environment. Table 2 shows a list of ports and services used to attack honeypots, real systems, and the proportion of systems attacked.

**Table 2:** The proportion of real and honeypot systems attacked using different ports across different systems available in the HackIT environment.

**Ports**	**Services running on the port**	**Proportion of honeypots attacked**	**Proportion of real systems attacked**
21/tcp	ftp	0.26	0.02
80/tcp	http	0.25	0.02
53/tcp	domain	0.00	0.18
25/tcp	smtp	0.17	0.00
135/tcp	msrpc	0.15	0.01
443/tcp	https	0.01	0.15
111/tcp	rpcbind	0.13	0.02
5,800/tcp	vncc http	0.00	0.15
3,306/tcp	mysql	0.00	0.12
8,080/tcp	apache	0.00	0.11
110/tcp	pop3	0.00	0.07
22/tcp	ssh	0.00	0.05
445/tcp	microsoft-ds	0.00	0.05
6,112/tcp	dtspc	0.00	0.03
139/tcp	netbios-ssn	0.00	0.03

### Influence of availability of subnets on real and honeypot system attacked

**Figure 5:**
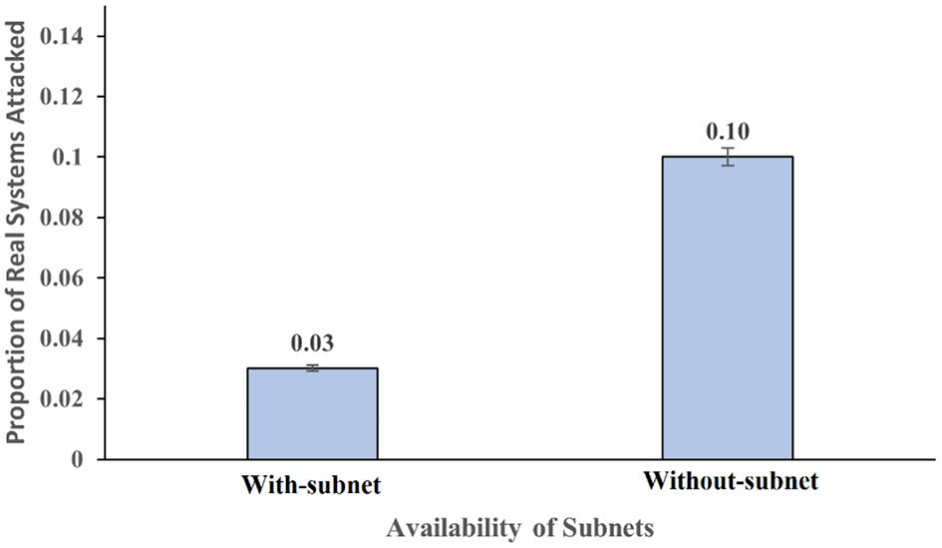
The proportion of real systems attacked across the presence and absence of subnets within a network. The error bars show 95% CI around the average estimate.

The availability of subnets significantly influenced the proportion of real system attacked (*F* (1,116) = 11.44, *p* =.001, η^2^ = 0.09). As shown in Figure 5, the proportion of real systems attacked was 0.03 in the presence of subnets within a network; however, the proportion of real systems attacked was 0.10 in the absence of subnets. The availability of subnets did not significantly influence the proportion of honeypot systems attacked (*F* (1,116) = 0.98, *p* =.32, η^2^ = 0.008). Overall, these results meet our expectations.

### Influence of port hardening on real and honeypot system attacked

**Figure 6:**
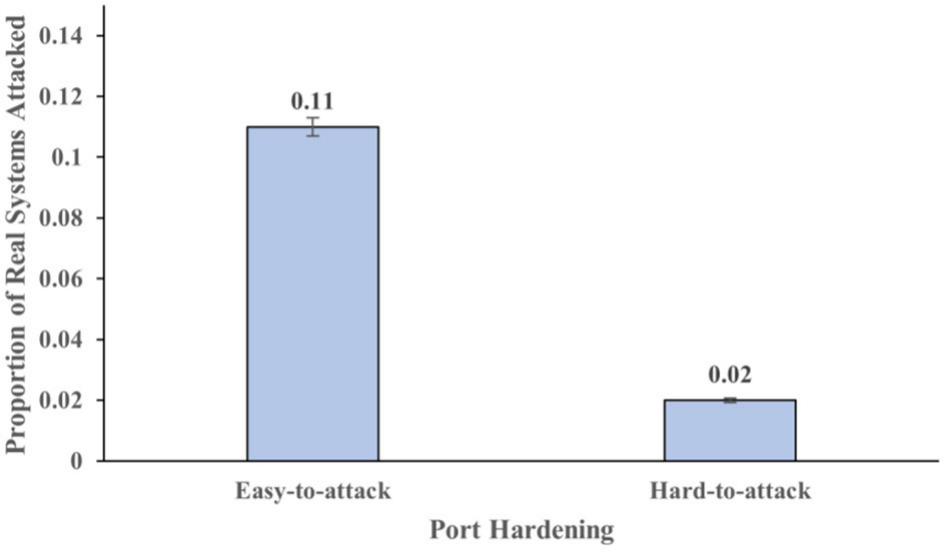
The proportion of real systems attacked across different levels of hardness. The error bars show 95% CI around the average estimate.

The hardening of ports of the systems also influenced the proportion of real system attacked (*F* (1,116) = 17.13, *p* < .001, η^2^ = 0.13). Figure 6 shows the proportion of real systems attacked across different levels of port hardness. The proportion of real systems attacked were 0.11 in the easy-to-attack conditions. In contrast, the proportion of real systems attacked were 0.023 in the hard-to-attack conditions. The hardening of ports of the systems did not significantly influence the proportion of honeypot systems attacked (*F* (1,116) = 2.85, *p* =.094, η^2^ = 0.024). These results also meet our expectations.

### Real and honeypot system attacked over time

**Figure 7:**
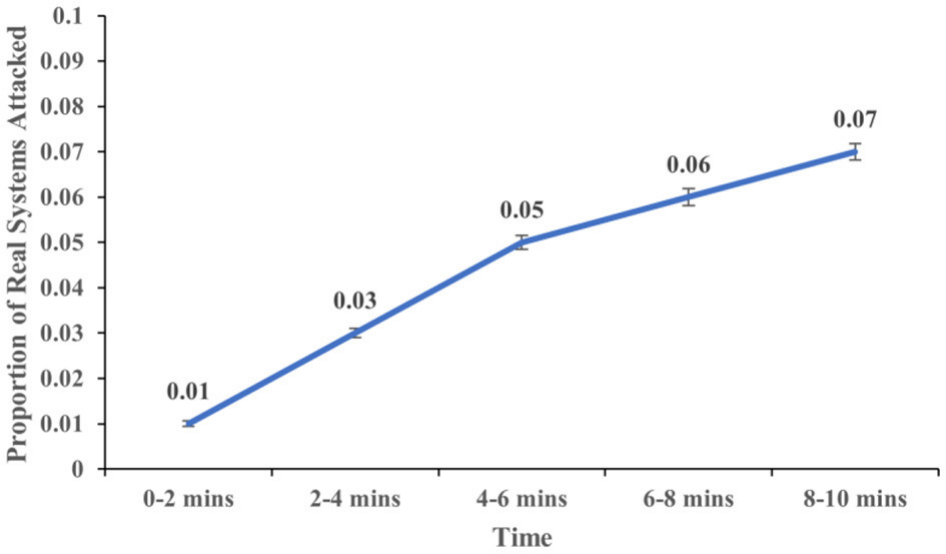
The proportion of real systems attacked across different time intervals. The error bars show 95% CI around the average estimate.

**Figure 8:**
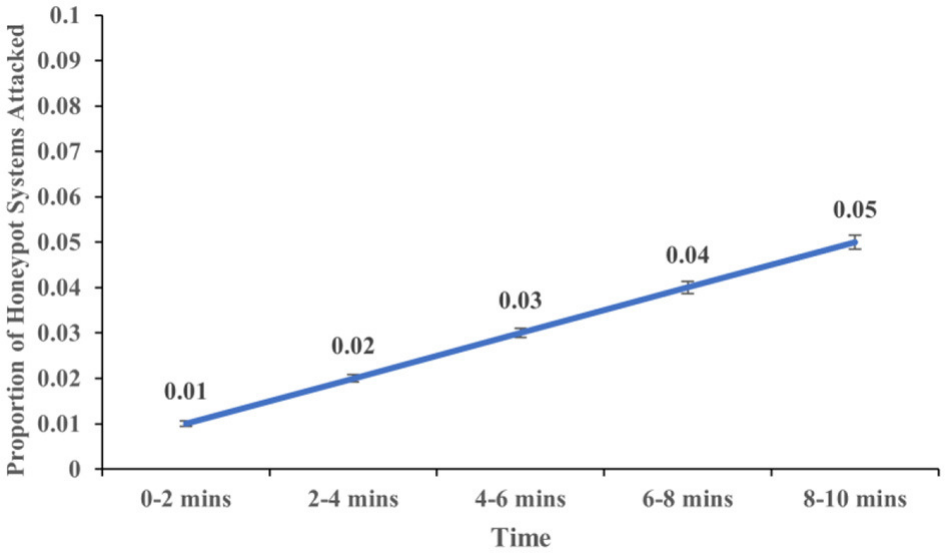
The proportion of honeypot systems attacked across different time intervals. The error bars show 95% CI around the average estimate.

The proportion of real systems attacked increased over time (*F* (4,113) = 5.937, *p* < 0.001, η^2^ = 0.174). Figure 7 shows the proportion of real systems attacked at different time intervals of 2, 4, 6, 8, and 10 min. Similarly, the proportion of honeypot systems attacked increased over time (*F* (4,113) = 12.547, *p* < .001, η^2^ = 0.308). Figure 8 shows the proportion of real systems attacked at different time intervals of 2, 4, 6, 8, and 10 min. Overall, these results meet our expectations.

### Influence of availability of subnets and port hardening on real and honeypot system attacks

**Figure 9:**
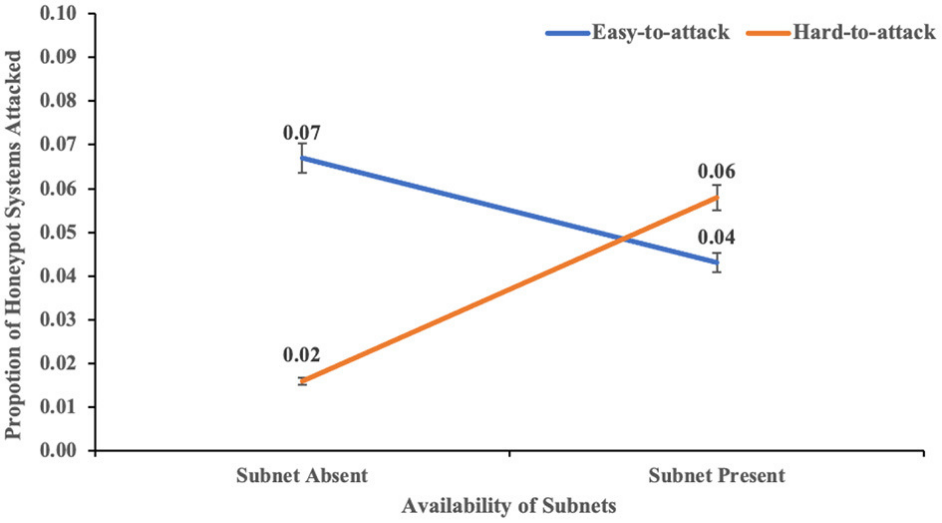
The proportion of honeypot systems attacked in with-subnet and without-subnet conditions across different levels of port hardness. The error bars show 95% CI around the average estimate.

The two-way interaction between the availability of subnets within a network and port hardening of the systems in the network on real systems attacked was not significant (*F* (1,116) = 2.98, *p* = 0.087, η^2^ = 0.25). However, the two-way interaction between the availability of subnets within a network and port hardening of the systems in the network on honeypot systems attacked was significant (*F* (1,116) = 7.014, *p* = 0.009, η^2^ = 0.057). This result indicated the influence of both availabilities of the subnet and port hardening on the proportion of honeypot systems attacked (see Figure 9). The *post-hoc* tests revealed that the proportion of honeypot systems attacked was significantly smaller in the hard-to-attack condition than the easy-to-attack condition in the absence of subnetting. However, there was no significant difference between the proportion of honeypot systems attacked in the hard-to-attack condition compared to the easy-to-attack condition in the presence of subnets. These results are also as per our expectations.

### Influence of availability of subnets on real and honeypot systems attacked over time

The interaction between availability of subnets and real systems attacked over time was not significant (*F* (4,113) = 1.67, *p* = 0.162, η^2^ = 0.056). Also, the two-way interaction between availability of subnets and honeypot system attacked over time was not significant (*F* (4,113) = 0.456, *p* = 0.768, η^2^ = 0.016).

### Influence of port hardening on real and honeypot system attacked over time

**Figure 10:**
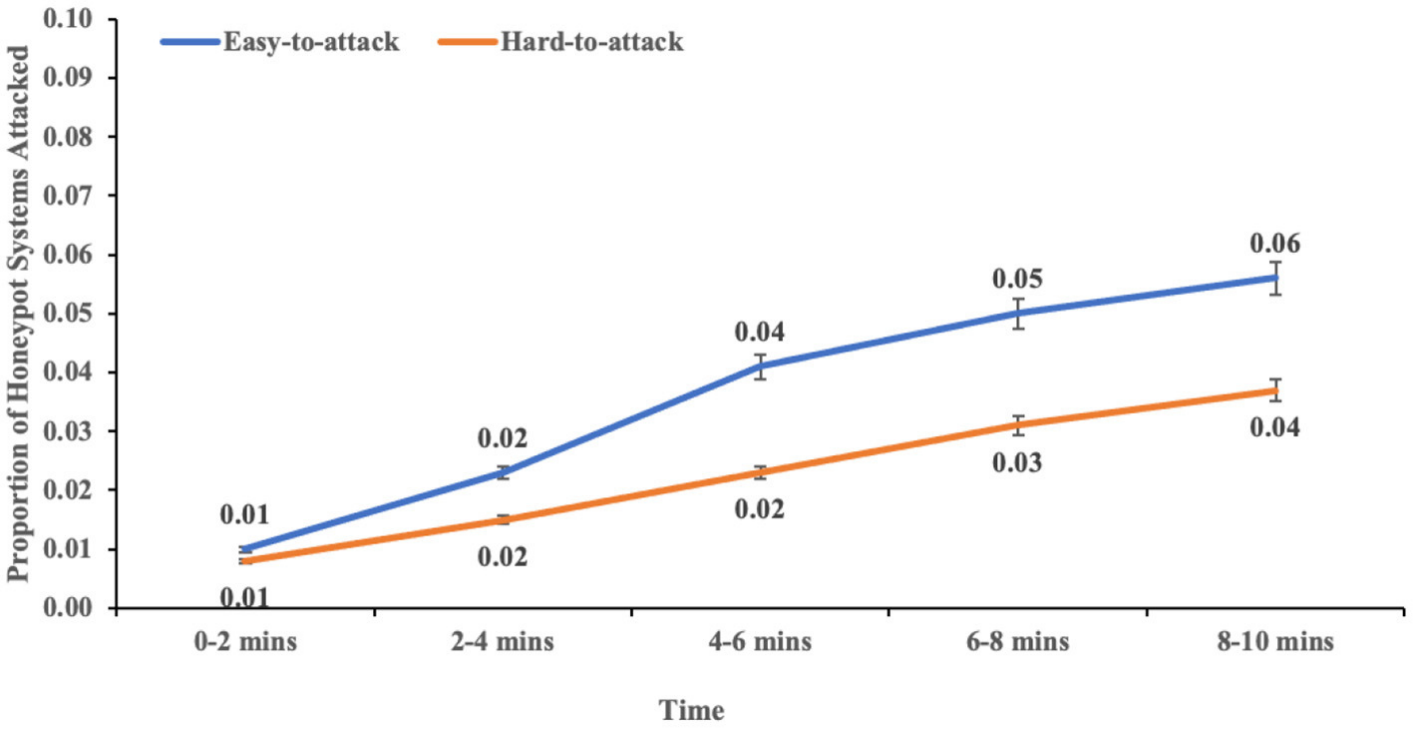
The proportion of honeypot systems attacked over time across different levels of port hardness. The error bars show 95% CI around the average estimate.

The interaction between port hardening and real systems attacked overtime was not significant (*F* (4,113) = 2.18, *p* = 0.076, η^2^ = 0.072). In contrast, the two-way interaction effect between port hardening and honeypot systems attacked over time was significant (F (4,113) = 2.795, *p* = 0.029, η^2^ = 0.09). This result indicated the influence of port hardening of the network systems on the proportion of honeypot systems attacked (see Figure 10).

### Influence of availability of subnets and port hardening on real and honeypot system attacked over time

**Figure 11:**
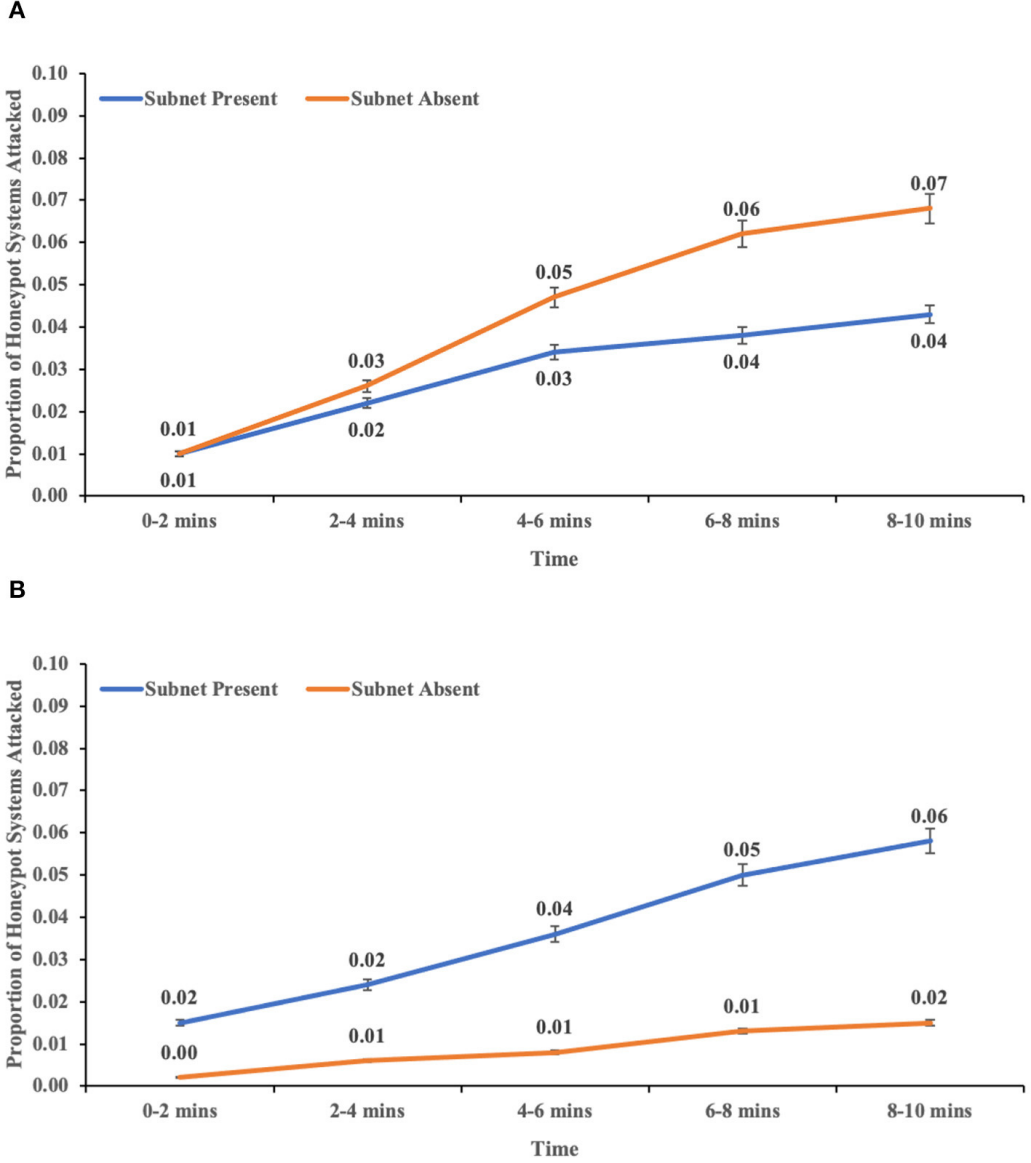
The proportion of honeypot systems attacked overtime across with-subnet and without-subnet conditions in **(A)** easy-to-attack conditions and **(B)** hard-to-attack conditions. The error bars show 95% CI around the average estimate.

The interaction between availability of subnets, port hardening, and real systems attacked overtime was not significant (*F* (4,113) = 0.316, *p* = 0.867, η^2^ = 0.011). However, the three-way interaction between availability of subnets, port hardening, and honeypot systems attacked over time was significant (*F* (4,113) = 2.757, *p* = 0.031, η^2^ = 0.089). This result indicated the influence of subnets' availability and port hardening on honeypot systems attacked over time (see Figure 11).

## Discussion and conclusion

Prior research in adversarial and behavioral cybersecurity experimented with deception techniques by introducing honeypots systems in the network (Furman et al., 2012; Addae et al., 2016; Caulkins et al., 2016). Researchers experimented with the effect of subnetting a network during a cyber-attack (Achleitner et al., 2017; Kelly et al., 2019). Researchers also investigated the effect of the amount and timing of deception and the influence of network size on an adversary's decision (Aggarwal et al., 2016; Katakwar et al., 2020). These experiments showed the importance of deception techniques, such as the addition of honeypots within a network, in preventing cyber-attacks and understanding adversary's attacking behavior to a certain extent (Aggarwal et al., 2016; Silic and Back, 2016; Sutton et al., 2019). However, little was known about the combined influence of subnets' availability and port hardening of systems on adversary's attacking behavior. Little was known about the combined effect of subnet availability and port hardness on adversary's attacking behavior over time. The main objective of this paper was to address these literature gaps.

Results revealed that the proportion of real systems attacked reduced in the condition where subnets were present. Similarly, increasing the hardness of ports of the systems within the network helped reduce the proportion of real systems attacked. Also, the proportion of real system and honeypot system attacked over time increased throughout the experiment's allotted time. Moreover, there was an influence of availability of subnets and port hardening on the proportion of honeypot systems attacked. There was also an influence of port hardening on the proportion of honeypot systems attacked over time. Furthermore, there was a combined influence of availability of subnets and port hardening on the proportion of honeypot systems attacked over time.

First, our results showed that a higher proportion of real systems were attacked when there was an absence of subnets within a network compared to subnets. This result agrees with the previous literature on subnetting benefits of a network in prevention from cyber-attacks (Jackson, 2015; Achleitner et al., 2017; Araujo et al., 2018; Kelly et al., 2019). The subnets' presence seemed to create a difficulty for the adversary to navigate through the network swiftly in search of real systems to hack. This difficulty may arise due to human adversaries' cognitive and memory limitations (Gonzalez et al., 2003; Dutt and Gonzalez, 2012).

Second, we found that a lesser proportion of real systems were attacked when port hardness was kept as hard-to-attack compared to easy-to-attack. The decrease in the proportion of real systems being attacked is likely because the hardened ports did not allow the adversary to access the system when attacked easily (Albanese et al., 2012; Dietz and Wallach, 2014). In hard-to-attack conditions, the probability of attacking a real system was 0.1, whereas, in the easy-to-attack conditions, the probability of attacking a real system was 0.9. This restriction and perhaps the limited attempts by adversaries on access to systems benefitted during the attack. This result agrees with the prior literature on system hardening (Dietz and Wallach, 2014).

Third, our results revealed that a higher proportion of real and honeypot systems were attacked overtime during the experiment. This increase in the proportion of real and honeypot systems being attacked is likely because adversaries gained familiarity with the network structure over time (Achleitner et al., 2017; Kelly et al., 2019). This increased familiarity facilitated the target systems and provided adversaries ample opportunity to attack. These findings agree with the prior literature (Achleitner et al., 2017; Kelly et al., 2019).

Fourth, results revealed a combined influence of subnets' availability and port hardening on the proportion of honeypot systems attacked. The increase in the proportion of honeypot systems attacked in the without-subnet and easy-to-attack (WoSE) condition compared to without-subnet and hard-to-attack (WoSH) stems from our prior findings that systems configured with hardened ports are not easily accessible by the adversary, thereby decreasing the chances of being attacked (La, 2014; Achleitner et al., 2017). This result agrees with the prior findings from the literature (La, 2014).

Moreover, there was an influence of port hardening of systems on the proportion of honeypot systems attacked over time (Dietz and Wallach, 2014). There was no difference in the proportion of honeypot systems attacked between the two different port hardness levels in the first 2 min of the experiment. However, the proportion of honeypot systems attacked started to increase significantly for easy-to-attack conditions compared to hard-to-attack conditions. Thus, the effect of port hardening seems to bind the adversary throughout the experiment (Albanese et al., 2012; Dietz and Wallach, 2014).

Furthermore, there was an influence of the availability of subnets and port hardening on the proportion of honeypot systems attacked over time. The important point to note here is that a higher proportion of honeypot systems were attacked in without-subnet conditions than with-subnet conditions in easy-to-attack conditions. In hard-to-attack conditions, a higher proportion of honeypot systems were attacked in with-subnet conditions than without-subnet conditions. One likely explanation for this phenomenon is that in with-subnet condition, there was a decreased visibility of systems present in the whole network, and the adversary tried to attack all possible systems within a subnet compared to moving to explore other subnets. In contrast, the without-subnet condition allowed the adversary to fully explore all the systems present within the network and make an informed decision about which system to attack.

This research has some implications for the adversarial and behavioral cybersecurity community. First, an implication from our results is that deploying subnets within a network containing honeypots indeed prevents real servers from being attacked by human adversaries. Second, it can also be implied that port hardening can help in reducing the risk of real servers being exploited in the presence of honeypots within the network. Third, intrusion detection systems with a response time of a few minutes can help prevent cyber-attacks.

A limitation of this research is that the results have been obtained from a laboratory experiment involving a simulation tool. Participants were recruited to participate as hackers from Amazon Mechanical Turk platform which offers little validated knowledge about the recruited participant sample and may suffer from selection bias. Although the experiment did not involve any real hackers, it was ensured that the recruited participants had an educational background or experience related to computer networking and network security. In fact, participants cleared a screening test on networking and cybersecurity with more than 70% accuracy before beginning their study. Although the real-world conditions could be different compared to the conditions stipulated in the lab some of the conclusions mentioned above are likely to hold in the real world. One of the reasons could be the lack of motivation for a hacker during the simulated cyberattack. However, the recruited participants were encouraged to attack as many systems as possible to maximize their rewards for a gift prize awarded to the top three scorers. Another reason for this expectation is because the HackIT tool was developed to simulate the real-world experience of using the command-line interface. The HackIT tool's user-experience was designed to replicate the look and feel of an actual command-line interface used to probe and attack webservers. Second, this research tried to reproduce the dynamics of a cyber-attack in the study using the HackIT tool: A network probe succeeded by a cyber-attack. Third, the ratio of real and honeypot systems within the network was kept low, which is likely to be similar to real-world situations to prevent cyber-attacks. Fourth, the knowledge of the network structure and the deception by honeypots was hidden from the adversaries as likely also hidden in the real world. This lack of information may not have given the adversary an advantage in the tool.

Numerous ideas can be taken forward from this research for future experimentation. First, the study can be replicated using the real-time network simulators such as GNS3 (Hassine and Hamou-Lhadj, 2014) and OMNeT++ (Varga and Hornig, 2008). It might be interesting to compare the results of real-time network simulation environments and HackIT environment in investigating the influence of subnetting and port-hardening on human decision during a cyber-attack. Second, researchers can explore various network topologies and compare their effectiveness in preventing a cyber-attack using real-time simulation tools such as HackIT. Third, the optimum value of port hardness can be investigated by varying the probability of success in attacking a system. Fourth, it might be interesting to develop computational cognitive models to predict human (or adversary) decisions during a cyber-attack using the HackIT tool with varied configurations and environment settings. Also, multi-player simulations can be developed to study the team behavior of adversaries during a cyber-attack. We plan to continue experimenting with some of these ideas in our ongoing research in adversarial cybersecurity.

## Data availability statement

The raw data supporting the conclusions of this article will be made available by the authors, without undue reservation.

## Ethics statement

The studies involving human participants were reviewed and approved by Ethics Committee, Indian Institute of Technology Mandi. The patients/participants provided their written informed consent to participate in this study.

## Author contributions

SU was responsible for the conducting the experiment and preparing the initial draft of the manuscript. PA and VD contributed with the inception of the idea for the experiment and revised the manuscript. All authors contributed to the article and approved the submitted version.
